# Heavy Metals in Biota in Delaware Bay, NJ: Developing a Food Web Approach to Contaminants

**DOI:** 10.3390/toxics7020034

**Published:** 2019-06-13

**Authors:** Joanna Burger, Nellie Tsipoura, Larry Niles, Amanda Dey, Christian Jeitner, Michael Gochfeld

**Affiliations:** 1 Division of Life Sciences, Rutgers University, 604 Allison Road, Piscataway, NJ 08854-8082, USA; jeitner@biology.rutgers.edu; 2 Environmental and Occupational Health Sciences Institute, Piscataway, NJ 08854, USA; gochfeld@eohsi.rutgers.edu; 3 New Jersey Audubon, 11 Hardscrabble Rd, Bernardsville, NJ 07924, USA; nellie.tsipoura@njaudubon.org; 4 Niles and Associates, 109 Market Lane, Greenwich, NJ 08323, USA; larry.niles@gmail.com; 5 Endangered and Nongame Species Program, Department of Environmental Protection, Trenton, NJ 08608, USA; Amanda.dey@dep.nj.us; 6 Rutgers Robert Wood Johnson Medical School and School of Public Health, Piscataway, NJ 08854, USA

**Keywords:** cadmium, lead, mercury, selenium, shorebirds, red knot, ruddy turnstone, sanderling, semipalmated sandpiper, blood, feathers, horseshoe crab eggs, *Limulus polyphemus*

## Abstract

Understanding the relationship between heavy metal and selenium levels in biota and their foods is important, but often difficult to determine because animals eat a variety of organisms. Yet such information is critical to managing species populations, ecological integrity, and risk to receptors (including humans) from consumption of certain prey. We examine levels of cadmium, lead, mercury, and selenium in biota from Delaware Bay (New Jersey, USA) to begin construction of a “springtime” food web that focuses on shorebirds. Horseshoe crab (*Limulus polyphemus*) eggs are one of the key components at the base of the food web, and crab spawning in spring provides a food resource supporting a massive stopover of shorebirds. Fish and other biota also forage on the crab eggs, and a complex food web leads directly to top-level predators such as bluefish (*Pomatomus saltatrix*) and striped bass (*Morone saxatilis*), both of which are consumed by egrets, eagles, ospreys (*Pandion haliaetus*), and humans. Metal levels in tissues were generally similar in algae, invertebrates, and small fish, and these were similar to those in blood of shorebirds (but not feathers). There was a significant direct relationship between the levels of metals in eggs of horseshoe crabs and mean metal levels in the blood of four species of shorebirds. Metal levels in shorebird feathers were higher than those in blood (except for selenium), reflecting sequestration of metals in feathers during their formation. Levels in feathers of laughing gulls (*Leucophaeus atricilla*) were similar to those in feathers of shorebirds (except for selenium). Selenium bears special mention as levels were significantly higher in the blood of all shorebird species than in other species in the food web, and were similar to levels in their feathers. Levels of metals in bluefish and striped bass were similar or higher than those found in the blood of shorebirds (except for selenium). The mean levels of cadmium, lead, and mercury in the blood and feathers of shorebirds were below any effect levels, but selenium levels in the blood and feathers of shorebirds were higher than the sublethal effect levels for birds. This is a cause for concern, and warrants further examination.

## 1. Introduction

Global changes, including increasing human populations, temperatures, sea level rise, and contaminants, have forced governments and communities to address these issues both locally and globally. While many issues need to be addressed globally (e.g., regulations for contaminants, protection of endangered and threatened species), the formulation of policy and regulations occurs at more local scales and requires information and data to support the regulations [1,2,3,4]. Collecting the necessary data often involves painstaking scientific studies at the local level, combined with larger ecosystem and regional studies. These studies often take the form of developing food webs, and there are two basic types: (1) examination of biota from plants to high trophic levels aimed at understanding the consumption patterns of individual species throughout the web (quantity and quality of foods eaten, nutrients, energy transfer) [5,6]; and (2) using species-based food webs to examine the movement of contaminants through food webs with the objective of understanding fate, transport, and effects [7,8,9,10]. Both have been improved by new techniques to examine foods consumed in relation to trophic level (e.g., stable isotope analysis [11]).

Government agencies, conservationists, and the public are interested in levels of contaminants in plants and wildlife that could prove detrimental to the organisms themselves, or to those that eat them, including people. While obtaining information on contaminant levels (and eventual effects) requires site-specific information from a wide range of species, many studies are either single species, or single year, or both. Developing food webs and associated contaminant webs is complicated, and often involves many years of study under conditions where both species composition and abundance, as well as contaminant levels, may be changing. For example, lead and cadmium have generally decreased over the last several decades [12,13], which has to be taken into account when examining the levels and effects in biota of food webs. Furthermore, levels of some contaminants may shift seasonally [14,15]. Levels also vary as a function of age and size in most animals, including fish and birds [9,16,17,18,19,20,21]. Generally, levels increase with age, although this is not always the case, and may not be true for all tissues [22,23]. Thus a series of food webs for different times of the year may be required, especially when there is conservation concern for threatened or endangered species, or for other species that are in decline.

In this paper we describe a food web for Delaware Bay, and examine cadmium (Cd), lead (Pb), mercury (Hg), and selenium (Se) levels in some of the biota for a springtime (May to early June) food web when shorebirds gather there in large numbers during their northward migration. We focused on shorebirds because they are transient and eat mainly crab eggs while staying at the Bay [24,25,26,27,28]. At other times and places their diet is more varied, making it more difficult to examine contaminants in their prey [29,30]. We were particularly interested in variations in blood (indicative of recent and local exposure) and feathers (indicative of exposure during feather formation on the wintering ground) of the shorebirds [22,31,32,33,34,35]. The Delaware Bay stopover thus provides a unique opportunity to examine some components of a food web where shorebirds are eating mainly one prey item, horseshoe crab (*Limulus polyphemus*) eggs. We also examined metal levels in some algae, the eggs and muscle of horseshoe crabs, other invertebrates, fish, and laughing gulls (*Leucophaeus atricilla*) [9].

Shorebirds are declining worldwide, and the species examined in this study are experiencing some of the most severe declines [36,37]. Red knot (*Calidris canutus rufa*) is currently listed as endangered in Canada and is threatened in the US [26,27]. Their decline is partly attributed to declines in the abundance and availability of crab eggs [26,38,39]. Similarly, semipalmated sandpipers (*Calidris pusilla*) have been declining along migration routes as well as in their South American wintering grounds [40,41,42].

Cd, Pb, and Hg were examined because they are key metal contaminants in oceans and bays [43,44], and Se because it can ameliorate the effects of Hg [45,46,47]. Coastal bays are particularly important to study. Metal levels may be higher in these ecosystems because of industrial runoff from rivers, as well as atmospheric deposition [48,49]. Metals are sequestered in the bottom sediments of estuaries, become incorporated into bacteria and other organisms living in the sediment, and are released by storms or strong tides. Feathers are easy to collect non-invasively and to store for long periods of time, and the metal levels often are directly related to levels of internal tissues [50]. Because many shorebird populations are declining [36,37], it is not always wise or permissible to collect them for the analysis of metals in internal organs.

## 2. Materials and Methods

### 2.1. Collecting Methods

**Figure 1:**
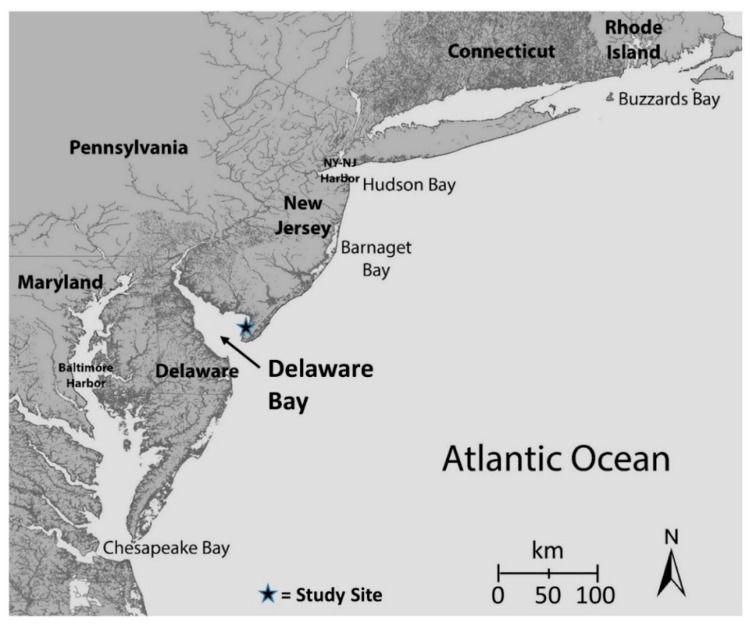
Map showing the location of Delaware Bay, between the states of New Jersey and Delaware. Arrow shows the approximate location of sampling.

For many years we have been examining heavy metals in a range of organisms (invertebrates, fish, birds) living in estuaries and bays along the Atlantic coast of North America. Under appropriate state and federal permits to collect invertebrates, fish, birds, and other wildlife, we have examined both temporal and spatial patterns of metals, particularly from Delaware Bay, Barnegat Bay, and the New York/New Jersey harbor (Figure 1) [9]. All methods were approved by the Rutgers University animal care committee (IACUC 92-036, renewed every three years) and conform to guidelines provided by the Ornithological Council (www.nmnh.si.edu/BIRDNET/GuideToUse). These guidelines have been formulated with consideration of animal welfare and research needs.

Samples were collected by a variety of methods in Delaware Bay. Algae, aquatic plants, and invertebrates were collected by hand, or with seine nets and traps [9]. Horseshoe crab eggs were collected from recently laid nests on spawning beaches; eggs were pooled from several nests [35,43]. Edible fish were collected from fishermen [51,52,53,54]. Shorebirds were captured by cannon net or mist nets. Blood was collected from wing veins in heparinized capillary tubes and refrigerated. A pinch of breast feathers was collected from each bird [33,35,55,56,57]. Blood was kept in a cooler until it was taken to the lab, where it was frozen for later analysis. There are negligible effects of blood sampling on migration and the reproductive performance of birds [58].

### 2.2. Chemical Analysis

Feathers were kept in envelopes at room temperature, but blood samples were frozen immediately for later analysis at the Environmental and Occupational Health Sciences Institute of Rutgers University. All tissues were analyzed by wet weight, except for the feathers (dry weight). Sample sizes for all tissues (and species) were greater than 15, to allow for individual variation.

Total Hg was analyzed by cold vapor atomic absorption spectrophotometry using a PerkinElmer FIMS-100 mercury analyzer (Waltham, MA, USA), of which about 85–90% is assumed to be methylmercury (MeHg) [59,60]. Other elements were analyzed by flameless, graphite furnace atomic absorption. Instrument detection limits were 0.02 ng·g^−1^ for Cd, 0.15 ng·g^−1^ for Pb, 0.2 ng·g^−1^ for Hg, and 0.7 ng·g^−1^ for Se. All specimens were analyzed in batches with known standards, calibration standards, and spiked specimens. Blanks, standard calibration curves, and spiked matrix specimens were used to monitor assay performance for all batches. All concentrations are expressed in ng·g^−1^ (ppb) wet weight for total metal in blood, and dry weight for feathers. Recoveries ranged from 85% to 103%. There were no batches with recoveries less than 85%. The coefficient of variation on replicate, spiked samples ranged up to 10%.

## 3. Results

### 3.1. Springtime Delaware Bay Food Web

**Figure 2:**
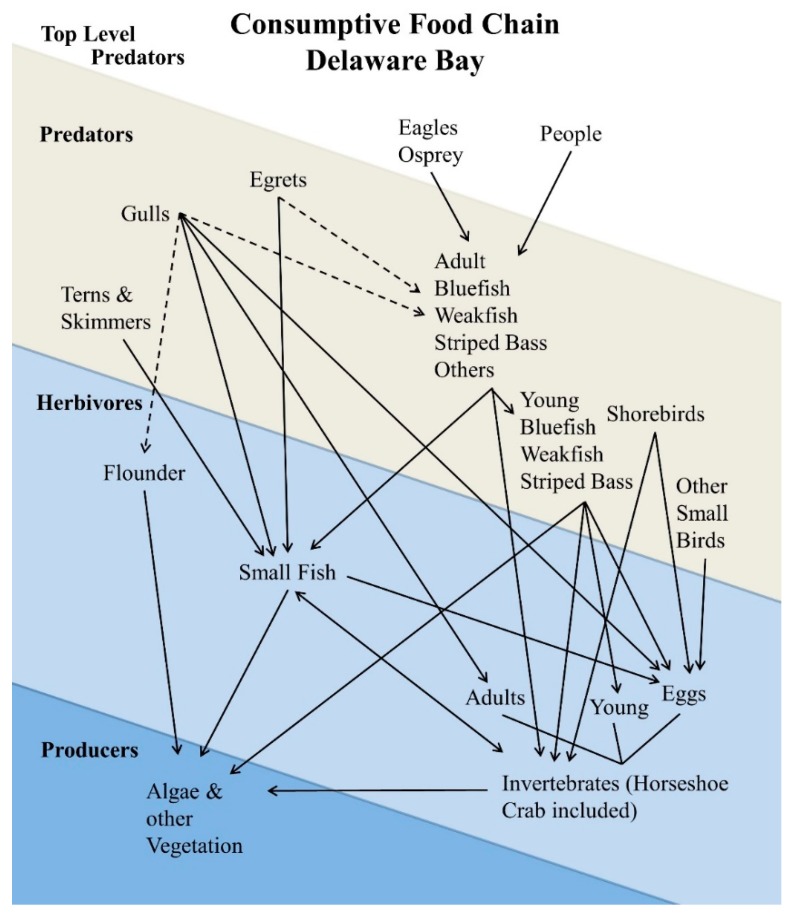
Schematic diagram of the consumption food web of Delaware Bay.

Horseshoe crabs are a keystone species on Delaware Bay, and form the basis of several food chains or webs (Figure 2) [9]. Within the Bay waters, many small fish and invertebrates feed on the crab eggs. During the spring migration, several species of migrant shorebirds stop at Delaware Bay to feed on the eggs of the crabs, while locally nesting willets (*Tringa semipalmata*) also forage on them sometimes (Burger, Unpubl obs.). Laughing gulls that nest on Atlantic coastal marshes gather in large numbers to feed on the crab eggs, sometimes competing with the foraging shorebirds for space [61]. While herring gulls (*Larus argentatus*) sometimes feed on crab eggs, they usually feed on the muscle of overturned and exposed crabs that have come up to spawn. Both gulls feed on a wide range of foods, including garbage. A number of small fish that feed on eggs are eaten by larger, edible fish, such as bluefish (*Pomatomus saltatrix*), striped bass (*Morone saxatilis*), and weakfish (*Cynoscion regalis*, [62]). Terns and black skimmers (*Rynchops niger*) also feed on small prey fish. In turn, eagles, osprey (*Pandion haliaetus*), egrets, and humans eat the bluefish, striped bass, and weakfish [9]. A simplified depiction of the food web is shown in Figure 2. The actual food web is more complex than this, with more nodes and species at each trophic level. Figure 2 gives an overall indication of the types of species and interactions.

### 3.2. Cd, Pb, Hg, and Se Levels

The levels of metals and Se in organisms on the food web are shown in Table 1. In general, levels were similar in algae, other plants, invertebrates (including horseshoe crabs and their eggs), and small prey fish. Levels of metals in the eggs of horseshoe crabs, however, were significantly lower than those in the muscle of adult crabs (Table 1). While feather and blood levels of Cd in shorebirds were similar to those in lower-trophic level organisms (e.g., within the same order of magnitude), and blood levels in shorebirds were similar (except for Se), the feather levels of Pb, Hg, and Se were higher (an order of magnitude higher, see Table 1). Bluefish and striped bass, both predatory fish, had similar levels of Cd, Pb, and Se as lower trophic-level biota, but higher Hg than lower trophic level biota. Laughing gulls had similar levels of Cd in their feathers as lower trophic biota had in their tissues, but the levels of Pb and Hg in feathers were similar to the levels in feathers of shorebirds.

Se levels, regulated in the body, were in the same order of magnitude for horseshoe crab eggs and muscle, flounder, bluefish, striped bass, and feathers of laughing gulls. All four species of shorebirds (except sanderling) had similar levels of Se in their blood and feathers (despite the fact that their feathers were grown on the wintering grounds in various part of South America).

### 3.3. Relationship between Levels in Horseshoe Crab Eggs and Shorebird Blood and Feathers

**Figure 3:**
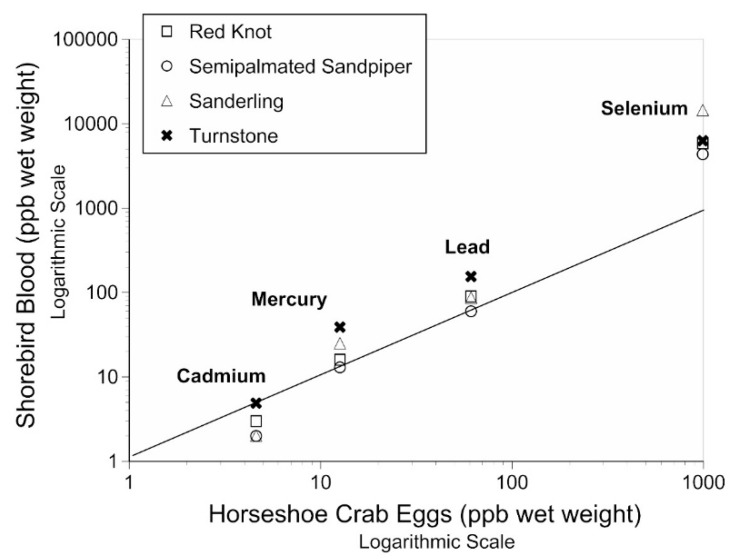
Relationship between the metals in horseshoe crab eggs and in the blood of four species of shorebird from Delaware Bay.

There is a clear, positive relationship between the levels of metals and Se in the eggs of horseshoe crabs and the blood of the four species of shorebirds (Figure 3). The relative relationship among the shorebirds is similar, with turnstones sometimes having a higher concentration of Hg than in the eggs of horseshoe crabs. Ruddy turnstones, a species whose metal levels were not previously examined by us, had the highest levels of Cd, Hg, and Pb (Figure 3). Se levels were higher in the blood of shorebirds than were the levels in the crab eggs.

### 3.4. Relationship between Feathers and Blood in Shorebirds

**Figure 4:**
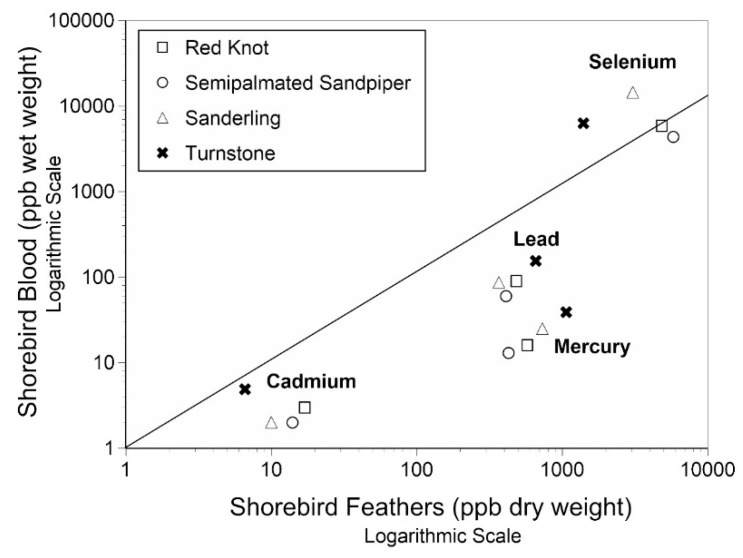
Relationship between levels of metals in the feathers and blood of four species of shorebird.

For all four shorebird species there was a close relationship between the levels of metals in their blood and feathers (Figure 4). That is, the values were clumped. There were no significant interspecific differences in metal levels for Cd, Pb, and Hg for knots, sanderling, and semipalmated sandpipers (*p* > 0.05 [35]). However, the levels of all metals in the blood of turnstones differed significantly from those of the other species (all chi-square values in this study were above 10.6, *p* > 0.01), although the differences were not great, except for Se. There were higher levels of Cd, Pb, and Hg in feathers for all four species, compared to levels in blood (Figure 4).

## 4. Discussion

This paper indicates the relationship between Cd, Pb, Hg, and Se for a range of organisms in a food web developed to represent springtime in Delaware Bay, New Jersey. Emphasis was placed on (1) levels of metals in lower trophic-level organisms, compared to higher level organisms; (2) the relationship between levels of metals in the blood of shorebirds compared with the levels in their prey (e.g., horseshoe crab eggs); and (3) the relationship between levels of metals in the feathers and blood in shorebirds. Each will be discussed below, along with possible methodological issues. Shorebirds were emphasized because of the importance of Delaware Bay in their annual cycle and population stability [24,26,27].

### 4.1. Methodological Issues

In this study, we relied on non-invasive sampling techniques (blood, feathers) in lieu of lethal techniques (collecting organ tissues) as an approach to studying the toxicokinetics of metals (absorption, tissue distribution, elimination) in threatened species (e.g., red knot) and in other shorebirds that are declining. One issue with this process is that we collected blood and feathers from the same individual birds [58], but did not collect internal tissues, as we did not want to impact populations. With species that have declining populations (such as the shorebirds in this study), it is not possible to euthanize shorebirds for this purpose (and indeed we would not want to do so). This means, however, that it is impossible to obtain blood and the other tissues (except feathers) from the same individual birds. To better understand toxicokinetics, however, it is essential to be able to correlate the levels in blood, feathers, and other internal tissues (liver, kidney, heart, brain [50,55]), but this should be done, perhaps, with gulls or other species whose populations are stable or increasing.

Another challenge was collecting prey foods that specific species were actually eating. This was simplified in the case of shorebirds during stopover in Delaware Bay, because they almost exclusively eat the eggs of horseshoe crabs [25]. The eggs of horseshoe crabs were collected for this study from the same beaches where the shorebirds were feeding, on the same days that they fed. Further, the data indicated a very high correlation between the metal levels in the eggs and in the blood of the shorebirds (see below).

While samples were collected during the three-week spring stopover on Delaware Bay, we did not collect during the first three to four days of shorebird arrival, to allow for absorption of metals and equilibration with blood. There is some indication that levels of metals in blood and feathers were more highly correlated when sanderlings were collected in mid-May compared to late-May for Hg [33]. This is intriguing, and needs further examination.

Finally, this paper represents the first stage of a trophic level analysis of biota in Delaware Bay. The Bayhas lost much of its commercial and recreational fishing because of collapses in fish populations, and it supports far fewer populations of migrating shorebirds, experiences industrial and agricultural pollution, and is facing additional habitat losses because of sea level rise [24,25,26,27,28,36,38,55]. To some degree, this paper is a call to gather more information on contaminant levels in more nodes on the food web so that the keystone species can be identified, the possible causal role of contaminants in species declines can be examined, and possible remedies can be identified and implemented. This requires not only studies of contaminant levels in a wider array of species, but using a range of field and laboratory techniques to examine biomethylation, trophic cascades, energy transfers, bioaccumulation, biomagnification, and DNA damage and biomarkers of oxidative stress [29,30,63,64,65,66]. The paper is intended to encourage these further studies.

### 4.2. Trophic-Level Relationships

Many previous papers have suggested that with each step in a food chain (or web), there is an increase (biomagnification) in the levels of metals (particularly Hg) in biota, which can result in higher metal levels at higher trophic levels [9,15,60,67]. In general, this was true for data presented in this paper (Table 1). However, there are some notable exceptions: (1) levels of Cd in the eggs of horseshoe crabs were an order of magnitude lower than the levels in muscle of adult crabs, but this was not true for the other metals; (2) Cd levels in shorebirds were similar to levels of lower trophic level biota, but the levels of Pb and Hg were higher in shorebirds; (3) Se levels at lower trophic levels were an order of magnitude higher than for the other metals; (4) Se levels were an order of magnitude higher in the blood and feathers of shorebirds than the other metals in shorebird tissues, and levels in shorebird blood and feathers were higher than Se levels at all other trophic levels; (5) flounder, a fish that eats vegetation, invertebrates, and small fish, had similar levels of most metals as were in its prey; and (6) levels of metals in bluefish were similar to other trophic levels, except for Se, which was lower than the levels found in shorebirds, but slightly higher than levels found in small prey fish.

The relatively low levels of Cd in all species and tissues examined contrasts with the data on bioaccumulation presented in this paper for the other elements. There is disagreement about whether Cd biomagnifies; some studies report higher levels in higher trophic levels, while others do not [68,69]. Unfortunately, there are few laboratory studies that examine the toxicokinetics of Cd and its role in the food chain [68]. Certainly, the data presented in this paper do not support bioaccumulation of Cd at higher trophic levels. It was not surprising that flounder had low and similar levels to the foods it eats in that flounder eat mainly low trophic-level foods, do not live long, and frequent bottom muds [62].

Levels of Se were higher than for Cd, Pb, and Hg in every species and tissue examined. Se is not examined routinely because, as an essential element, its levels are regulated in the body and thus expected to be relatively stable [70]. Also, Se has a narrow range of non-toxicity within the body, between a deficiency state and a toxic level [12,70,71]. The levels of Se in feathers of shorebirds were higher than those in laughing gulls, which might be expected as the gulls eat fish and other invertebrates in addition to horseshoe crab eggs (examining levels of Se in blood of the gulls would be instructive). Moreover, Se levels in the blood of shorebirds were quite high compared to the other metals. For the other metals examined in this paper, the levels in feathers were an order of magnitude higher than that in the blood, while the levels of Se in the blood and feathers of shorebirds were similar, except for sanderlings, where Se was nearly five times higher in their blood than in their feathers (and three times higher than in the blood of the other shorebird species). Since all four species primarily eat horseshoe crab eggs while in Delaware Bay, the difference among shorebird species is unexpected. It suggests that the toxicokinetics within sanderlings must be different; it appears they may be bioaccumulating Se at a greater rate than the other shorebird species. This finding needs to be examined in greater detail, over several years.

### 4.3. Metal Levels in the Eggs of Horseshoe Crabs and Blood of Shorebirds

The levels of metals in the blood of shorebirds significantly correlated with those in their primary food—Horseshoe crab eggs. It is unusual to have birds eat only one food item over a several-week period, providing a unique opportunity to examine this relationship. Still, there are some differences: (1) Cd levels in shorebird blood were similar (turnstones) or slightly lower than in crab eggs; (2) Hg, Pb, and Se were equal or slightly higher in shorebird blood than in crab eggs; and (3) turnstones generally had the highest levels of metals in blood compared with the other shorebird species.

The slightly higher accumulation of metals in the blood of turnstones, compared with the other species, might simply reflect internal toxicokinetics. This could, however, also reflect some differences in foraging method and habitat use. That is, turnstones often forage by digging up crab egg clutches that are intact, while other species all forage exclusively for eggs that have been dug up by other crabs spawning, or by erosion or wave action. Since the eggs of a clutch stick together, it is possible that females exude a substance that contains contaminants that bind the eggs, but this requires further study. Eggs washed ashore in the surf are loose and would be without any exudate.

The tight relationship between the metals in the crab eggs and the blood of shorebirds suggests that blood should be examined in other birds (and fish, if possible) that are foraging for the crab eggs. Herring gulls, for example, forage for the muscle of adult crabs overturned by the surf while spawning, as well as other foods, and the levels of Se in their blood may be different than that of the shorebirds.

### 4.4. Relationship of Metals in the Blood and Feathers of Shorebirds

One of the findings that is key to understanding exposure and effects is being able to understand the relationship between tissues, so that examining one or more tissues would result in being able to predict levels in other tissues. It is important to know whether there is a relationship between the levels of metals in feathers and blood, and whether that is similar among shorebird species. The ratio of levels of metals in blood/feathers did not differ significantly for Cd (0.13–0.17), Pb (0.15–0.24), or Hg (0.03 for all species) for knots, sanderlings, and semipalmated sandpipers [35]. In a study of the internal tissues of semipalmated sandpipers [55], the ratios of feathers to liver, muscle, and brain were determined. These ratios could be used with the current feather levels of the other shorebird species to determine the metal levels in brain and liver, for example, to determine whether there are adverse effects. The ratios varied from 0.06 (Hg) to 0.14 (Pb), to 0.18 (Cd) for brain/feathers [55]. The ratio was lowest for Hg, perhaps indicating a strong blood/brain barrier for Hg, which is a neurotoxin [72,73]. Significantly more research on the relationships between tissues that are easily collected non-lethally (feathers, blood) and internal tissues is needed before specific effects can be determined.

### 4.5. Effects Levels of Metals for Shorebirds

It is instructive to determine whether either blood or feather levels in shorebirds indicates any cause for concern, especially given that shorebirds are declining globally [36,37,42]. Delaware Bay has historically had high industrial activity, with petroleum refineries, transport facilities, shipyards, steel manufacturing, and chemical and plastic industries, among others. It is highly urbanized and has historically used agricultural chemicals [74,75]. In general, levels of metals have been declining over time, partly due to regulations on emissions and effluents. For example, the levels of Pb and Cd in the environment have declined due to regulations on Pb in gasoline and Cd in batteries [76]. Temporal trends in Hg levels are less clear, partly because of the tension between historic increases in regulations, more recent decreases in regulations, and increases in the building of coal-burning power plants in China [73,76]. Temporal trends in some metals in shorebirds have declined over time [77,78], although Hg has remained the same and Se has increased [9].

The levels of Cd in this study were low compared with levels summarized in the literature for feathers [50] and blood [9,79]. Although few laboratory experiments have been conducted on Cd in birds, Eisler [80] reported an adverse effects level at 10,000 ppb in birds. The Cd levels in shorebird blood and feathers (highest mean Cd levels were below 20 ppb) were well below the effects levels. Similarly, the levels of Pb in the blood and feathers of shorebirds were well below any effects levels. The adverse effects level for Pb is 2000 ppb in blood [81]; 4000 ppb in feathers also results in sublethal adverse effects [82,83]. The highest mean Pb level in feathers was 484 ppm, and blood was well below that (mean of 155 ppm). In humans, current evidence suggests that no adverse effect level or toxic threshold has been established, meaning the even very low levels Pb is considered harmful [13]. Pb is unique among metals in that the US Environmental Protection Agency has not established a reference dose (EPA *Integrated Risk Information System* [IRIS] data base). Few very low level experiments on the effects of Pb on birds have been conducted, and there has not been a no observable effect level (NOEL) established for either Cd or Pb in birds.

Hg, usually the contaminant of most concern for marine organisms [44], can affect the behavior, physiology, and reproductive success of birds [59,72,82]. The highest mean level of Hg in feathers of shorebirds in this study was 730 ppb (sanderlings), and in blood it was 39 ppm. Both are well below the adverse effect level of 5000 ppm [72], although Jackson et al. [83] found that lower levels resulted in adverse effects in wrens (*Thryothorus ludovicianus*). The actual toxic effects of Hg on shorebirds, however, may be lower than in birds generally because of their long evolution in marine environments.

Se levels are seldom examined in birds, except in specific instances (e.g., Kesterson [84]). However, given the apparent ability of Se to modify the adverse effects of Hg [45,46,47], it is important to examine selenium in future studies. Levels of Se in feathers that are associated with adverse effects in birds range from 1.8 ppm for sublethal effects, to 26 ppm for lethality [9,70,85]. The mean levels of Se of shorebirds from this study were below the lethality level for both feathers (highest mean of 5.8 ppm in semipalmated sandpiper) and blood (highest mean of 14.5 ppm in sanderling). Thus, the mean se levels in shorebirds from Delaware Bay are well below the lethal levels, but they are not below the sublethal effects levels. This suggests that it would be useful to conduct laboratory studies into the effect of these levels of Se on shorebirds, and to do so in relation to Hg levels.

The relationship between Hg and Se, and the possible adverse effects of either, bears further examination, both in field observations and laboratory studies. Ralston and others have postulated that Se ameliorates the effect of Hg [45,46,47], and that a molar ratio of 1 or above protects from the adverse effects of Hg. They proposed that the adverse effects of Hg in fish could be prevented by consuming fish with excess Se [86]. Ralston and Raymond [47] suggested that Hg’s neurotoxicity is characterized by its disruption of Se biochemistry. They proposed that an Hg-dependent sequestration of Se causes an irreversible inhibition of selenoenzymes that is primarily responsible for the characteristic effects of Hg toxicity. This requires further study in various tissues from a range of organisms, including fish and birds.

### 4.6. Effects in Other Biota

The food web in Delaware Bay is complex, even more so than has been illustrated in Figure 2. Clearly, one food chain is from horseshoe crab eggs through small fish to larger fish, terns, skimmers, gulls, egrets, eagles, and ospreys. The large-sized bluefish, weakfish, and striped bass are eaten by eagles, osprey, and people. Shorebirds are no longer eaten by people living in the United States, but they are hunted and eaten by people in South America, as well as in some Arctic regions [87,88,89,90]. Contaminants in lower trophic-level organisms can clearly have an impact on higher trophic levels (predators and top-level predators, Figure 2). Although not the main topic of this paper, the relative importance of the levels of metals in other species merits commenting on.

The contaminant of most concern for marine organisms is Hg in the form of methylmercury (MeHg) [73]. The dietary levels known to cause ill effects in vertebrate biota range from 0.5 to 5 ppm (or 500 to 5000 ppb [72]), although they may be as low as 0.1 ppm in sensitive mammals and 0.2 ppm in sensitive birds [91]. As is always the case, it is difficult to know whether any given species falls into the “sensitive” category. In the present study, levels of Hg in small prey fish, the food for larger predatory fish, ranged from 20 to 50 ppb, which is well below the effect level. However, bluefish and striped bass had Hg levels of 300 and 740 ppb in their muscle, which are within the range where sensitive higher-trophic level predators (such as even larger fish, predatory birds such as osprey, or scavengers) might be adversely affected. Osprey, the only raptor that eats only fish, likely evolved with the relatively high Hg levels in marine fish, and so it is unclear at what level they suffer adverse effects. Some oceanic marine birds, for example, are able to demethylate MeHg, reducing its effects [14].

Striped bass (at a mean of 740 ppb, or 0.7 ppm) exceeds the action of 0.5 ppm limit for human consumption (some US states and in Europe), although it is below 1.0 ppm (US action level [73]). The US Environmental Protection Agency [92] has set the freshwater criterion of 0.3 ppm Hg in fish tissue, based on its reference dose to protect human consumers. Thus the average Hg levels in both bluefish (averaging 300 ppb (0.3 ppm) and striped bass (averaging 740 ppb, 0.7 ppm) are within the range to adversely affect people who eat these fish regularly [93,94]. For people eating shorebirds in either South America or the Arctic, shorebirds may pose a threat if levels in tissues are high. There was, for example, over half as much Hg in liver as feathers for semipalmated sandipipers [55], which would certainly fall within the 0.5 ppm action limit for Hg.

Se is the other element of possible concern in the food chain described for Delaware Bay, largely because Se levels were comparatively high in some species. In general, levels of Se in food of 1000 ppb are toxic to some wildlife that consume them [70]. The mean level of Se in semipalmated muscle was 1510 ppb [55]. Levels of Se in blood and feathers were similar among shorebirds in the present study, which suggests that Se levels may be similar in the muscle of different shorebird species, potentially posing a risk to predators that consume them.

## 5. Conclusions

The data presented are only the beginning of constructing a complex food web, with associated contaminant levels, for Delaware Bay, New Jersey. Nonetheless, they indicate that metal levels are similar among algae, small invertebrates, and small fish. Levels of metals in the blood of shorebirds reflect the levels in horseshoe crab eggs, their primary food while stopping over during spring migration. Levels in shorebird feathers were considerable higher, reflecting sequestration during feather formation. Sequestering metals in feathers is a method of ridding the body of these contaminants. Se levels, however, were significantly higher in blood and feathers of shorebirds than for all other nodes on the food web presented. The level of Se in blood of sanderlings was nearly five times higher than the level in their feathers, and three times higher than in the blood of the other shorebird species. Further, although Se levels in blood and feathers of shorebirds were below the lethal effects level for Se, they were above the known sublethal effect level in birds, which bears further examination. Laboratory studies may be necessary to determine whether Se levels in the blood of shorebirds presents a potential risk, and whether the relationship between Hg and Se ameliorates the toxicity of both. More work is required to construct the complex food web and associated contaminants for even the springtime. Further field and laboratory studies are needed to fully explore the trophic food web of Delaware Bay, including the potential sublethal effects on biota and cascading effects.

## Figures and Tables

**Table 1:** Metal levels in some key organisms of the food chain in Delaware Bay, New Jersey. Given are range of means (or mean ± SE) (after Burger and Gochfield [9,52]; Tsipoura et al. [33]; Burger et al. ([35,53,56,57], unpublished data); Burger and Tsipoura [43]; Burger [51]; and Gochfeld et al. [54]).

Trophic Level			Cadmium	Lead	Mercury	Selenium
Algae and plants (ppb ww) ^b^			^a^	65–80	4–6	^a^
Horseshoe Crabs						
	Eggs (ww) ^c^		0.4 ± 0.2	25 ± 5	1 ± 0.1	996 ± 137
	Muscle (ww)		37 ± 6	41 ± 4	57 ± 5	876 ± 39
Other Invertebrates			4–30	22–32	11–32	160–230
Small prey fish (whole)			2–5	20–300	20–51	411–577
Flounder			10 ± 2	60 ± 10	150 ± 10	360 ± 100
Shorebirds						
	Red Knot					
		Feather	17 ± 2.4	484 ± 67	576 ± 105	4835 ± 432
		Blood	3 ± 0.7	90 ± 12	16 ± 3.1	5873 ± 573
	Turnstone					
		Feather	7 ± 1.4	658 ± 93	1065 ± 208	1398 ± 176
		Blood	5 ± 0.8	155 ± 27	40 ± 6.7	6294 ± 785
	Sanderling					
		Feather	10 ± 2.6	367 ± 52	730 ± 109	3057 ± 781
		Blood	2 ± 0.7	87 ± 14	25 ± 5.3	14500 ± 2300
	Semipalmated Sandpiper					
		Feather	14 ± 2.7	411 ± 46	428 ± 58	5802 ± 562
		Blood	2 ± 0.5	60 ± 11	13 ± 3.3	4422 ± 470
Bluefish			6 ± 2	60 ± 10	300 ± 30	510 ± 40
Striped Bass			0.6 ± 0.3	16 ± 4	740 ± 20	290 ± 20
Laughing Gull (feathers)			1 ± 2	510 ± 35	650 ± 45	910 ± 48

^a^ = Not available; ^b^ = range of means for different species; ^c^ = mean ± standard error.
